# Spontaneously directed loop extrusion in SMC complexes emerges from broken detailed balance and anisotropic DNA search

**DOI:** 10.1093/nar/gkaf725

**Published:** 2025-08-01

**Authors:** Andrea Bonato, Jae-Won Jang, Do-Gyun Kim, Kyoung-Wook Moon, Davide Michieletto, Je-Kyung Ryu

**Affiliations:** Department of Physics, University of Strathclyde, Glasgow G4 0NG, United Kingdom; Interdisciplinary Program in Computational Science, Seoul National University, Seoul 08826, South Korea; Department of Physics and Astronomy, and Institute of Applied Physics, Seoul National University, Seoul 08826, South Korea; Department of Physics and Astronomy, and Institute of Applied Physics, Seoul National University, Seoul 08826, South Korea; School of Physics and Astronomy, University of Edinburgh, Peter Guthrie Tait Road, Edinburgh EH9 3FD, United Kingdom; MRC Human Genetics Unit, Institute of Genetics and Cancer, University of Edinburgh, Edinburgh EH4 2XU, United Kingdom; Interdisciplinary Program in Computational Science, Seoul National University, Seoul 08826, South Korea; Department of Physics and Astronomy, and Institute of Applied Physics, Seoul National University, Seoul 08826, South Korea

## Abstract

DNA loop formation by structural maintenance of chromosome (SMC) proteins, including cohesin, condensin, and the SMC5/6 complex, plays a pivotal role in genome organization. Despite its importance, the molecular mechanism underlying SMC-mediated loop formation, particularly how these complexes achieve persistent directionality (rectification) while minimizing backward steps during the formation of large loops, remains poorly understood. Here, we use atomic force microscopy (AFM) and computational simulation to uncover a key geometric feature of the yeast condensin SMC complex enabling rectified loop growth. Using AFM, we demonstrate that the hinge domain of yeast condensin exhibits a directional bias, extending orthogonally to the bound DNA and sampling an anisotropic region of space around the protein complex. By accounting for the geometric constraint on the hinge-mediated DNA-capture step, we computationally show that loop growth can spontaneously self-rectify. In contrast, an SMC model with broken detailed balance and isotropic search instead exhibited substantial loop shrinkage and random-walk-like behaviour. These findings reveal an overlooked, and potentially broadly conserved, anisotropic DNA capture mechanism through which SMC complexes form and stabilize DNA loops *in vivo*, in turn providing novel insights into the physical principles governing genome organization.

## Graphical Abstract

**Graphical Abstract:**
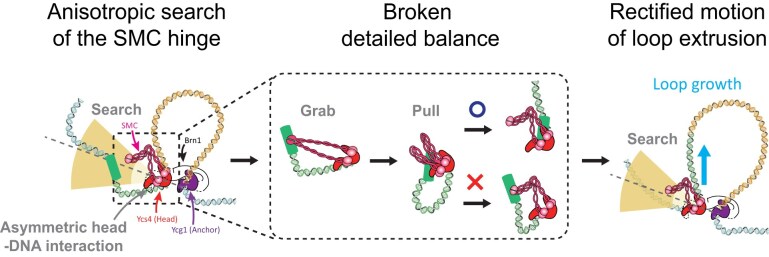

## Introduction

**Figure 1.**
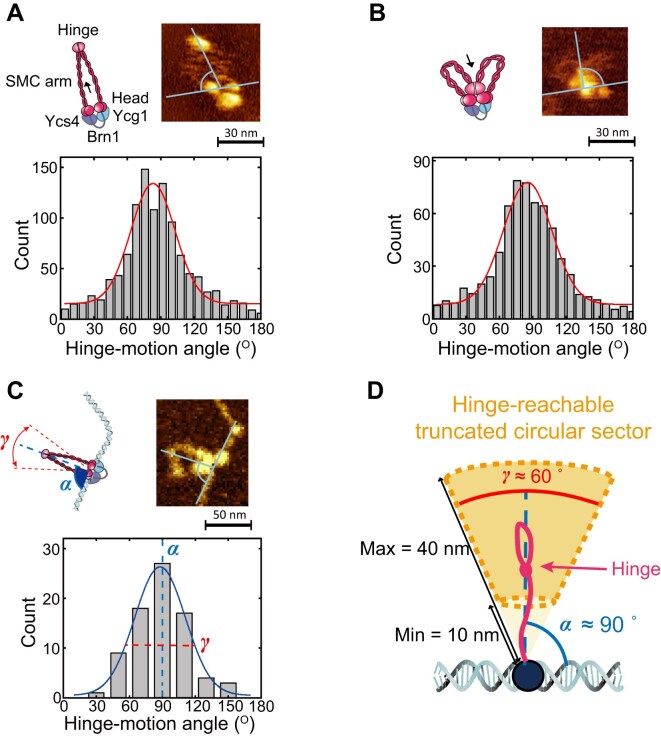
AFM imaging reveals that the hinge prefers to lie within a truncated circular sector orthogonal to the SMC heads bound DNA segment. (**A** and**B**) Angle distributions of (A) hinge-releasing (mean ± s.d. = 83 ± 29°; *N* = 1096) and (B) hinge-engaging movements (mean ± s.d. = 86 ± 44°; *N* = 1107) respect to the line connecting two SMC heads, as observed by HS AFM. (**C**) Distributions of the angle between the hinge and the central position of the globular domain with respect to tangential DNA line analyzed by dry-AFM images (mean ± s.d. = 88 ± 23°; *N* = 79). (**D**) Schematic representation of the truncated cone defining the hinge-reachable region.

Loop formation by structural maintenance of chromosome (SMC) proteins such as cohesin, condensin, and the SMC5/6 complex has emerged as a universal organizing principle of chromosome structure and function [1–12]. In eukaryotes, cohesin mediate the formation of “topologically associating domains” (TADs) during interphase [13–16], while condensin is essential for organizing mitotic chromosomes [17, 18]. An SMC complex has a ring-like architecture composed of an SMC dimer and an intrinsically disordered kleisin subunit. The SMC dimer is formed through the “hinge” region, with the kleisin subunit binding asymmetrically to two SMCs—one at the neck and the other at the head (Fig. 1A) [19]. ATP binding to the heads induces asymmetric dimerization of the ATPase heads [20]. Additionally, two HEAT repeat subunits (Ycg1 and Ycs4 in yeast condensin) are asymmetrically associated with the kleisin subunit (Brn1 in yeast condensin), creating a structure reminiscent of clothes hanging on a clothesline [21, 22]. Despite extensive structural and mechanistic studies, conflicting evidence persists regarding the exact topology and mechanics necessary for achieving systematic DNA loop growth, as observed in tethered DNA experiments [7, 8, 10].

One of the unsolved problems lies in understanding the origins of directed (or “rectified”) loop extrusion, i.e. how do SMC complexes avoid substantial backward steps during the formation of large loops, as this would induce loop shrinkage [23–28]. Understanding the origins of this directed (or “rectified”) loop growth is different from understanding the mechanics of ATP-driven protein stepping [24] or the origin of the “left-right” directionality bias, which is mostly due to the 5′→ 3′ or 3′→ 5′ direction of the heads with respect to DNA [22, 29]. More specifically, broken detailed balance is a necessary component to yield loop growth, indeed if the protein did not break time reversal symmetry, loops would be expected to eventually shrink back; however, breaking detailed balance through ATP consumption is not sufficient to guarantee a rectified process. Indeed, in analogy with other active systems, rectified motion typically arises when ATP hydrolysis is coupled to a chiral element that disrupts spatial symmetry [30]. In other words, rectified motion cannot be explained simply by the fact that SMCs consume ATP and instead require stronger conditions.

Recent work suggests that SMCs use conformational changes between a hinge-released state—where the hinge is extended away from the heads—and a hinge-engaged state—where the hinge is in proximity of the heads, in order to drive its motion in a “scrunching,” and ATP-dependent, fashion [12, 25, 28, 31–34] (Fig. 1A and B). The scrunching model predicts that following dimerization of the SMC heads (due to ATP-binding), the coiled-coil arms fold to bring the hinge closer to the heads. Following ATP-hydrolysis, the heads are released, and the hinge extends again [12, 25, 35]. During this step, the hinge may search for a 3D proximal (but not necessarily 1D contiguous) DNA segment to grab and subsequently brings it close to the heads in the following ATP-binding step [25, 36]. This model includes a cycle between states of the protein that is irreversible, thus requiring ATP consumption to break detailed balance. Whilst this model can elegantly explain the bypassing of other SMCs [28, 37, 38] and large roadblocks [39, 40], it cannot explain directed motion. Indeed, during the search step, there is no guarantee that the hinge will grab onto a DNA segment outside of the extruded loop. If the grabbed DNA segment is located on the inside the extruded loop, the SMC step would reduce the overall loop size. Thus, even within the scrunching model, explaining the observed rectified loop extrusion remains an outstanding problem in the field of SMC-driven DNA organization.

Alternative mechanisms, including the “reel-and-seal” and “DNA-segment capture” models, also emphasize the hinge’s role in targeting DNA during loop extrusion and ATP-cycle driven arrangement of the heads and non-SMC subunits [11, 34, 41, 42]. However, these models share the challenge of explaining how directional bias is maintained during the search and capture steps. This raises a broader question: what are the minimal structural and energetic requirements for persistent, rectified motion in a protein–DNA complex? More broadly, it is of generic physical interest to understand what are the minimal ingredients that generate persistent motion in the protein–DNA complex that consumes ATP.

In this paper, we investigate the origin of the rectified motion using yeast condensin SMC complex, atomic force microscopy (AFM) [12, 43] and molecular dynamics (MD) simulations. Our AFM experiments show that the hinge exhibits a geometric bias, preferentially extending orthogonally to the bound DNA segment. This observation led us to hypothesize that a geometric (angular) constraint on the hinge extension could create a spatial bias in the DNA segment capture during loop formation. To test this hypothesis, we incorporated this geometric constraint into computational models, simulating loop extrusion with an anisotropic search mechanism and asymmetric head-to-DNA alignment. Remarkably, these simulations demonstrated that loop extrusion can spontaneously self-rectify, even in the absence of explicit directional cues. Importantly, we demonstrate that ATP hydrolysis is not sufficient by itself to generate rectified loop growth and requires a broken spatial symmetry in the DNA-capture process.

Our work thus suggests that an anisotropic DNA capture due to a structural preference of the hinge to capture DNA orthogonally to the heads-bound DNA segment could explain the rectified loop growth observed *in vitro* on tethered DNA and at the same time explain a number of other puzzling experimental observations, such as in trans DNA capture [44], obstacle bypassing [40], and Z-loops [38].

## Materials and methods

### Condensin purification

We used the same protocol as previously reported [12] for the purification of *Saccharomyces cerevisiae* condensin holocomplexes with all the subunits.

### Liquid-phase high speed-AFM (HS AFM) imaging

Wild-type condensin holocomplex (at concentration of 2 nM) was deposited onto a freshly cleaved mica surface using a buffer composed of 20 mM Tris–HCl (pH 7.5), 50 mM NaCl, 2.5 mM MgCl_2_, 2.5 mM Dithiothretiol (DTT), along with 1 mM ATP (Fig. 1A and B, and Supplementary Fig. S1) [12]. After 10 s deposition, the sample surface was rinsed with the same buffer. The condensin sample was imaged using the HS AFM developed by RIBM. This was achieved by using either Nanoworld SD-S-USC-f1.2-k0.15 cantilevers (with a tip radius of 2 nm; spring constant, *k* = 0.15 N/m; and frequency, *f* = 1.2 MHz). During the imaging process, a scan size of 100 nm × 100 nm was typically used, along with 100–150 scan lines and frame rates ranging from 1 to 10 Hz. Typical images are shown in Supplementary Fig. S1A. A full movie of the experiment is shown in the Supplementary Movie.

The obtained data were processed using a Python script to reconstruct both movie files and images. Within this framework, a Python script was used to measure the angle of hinge-releasing and hinge-engaging movements. To define the transition state between hinge-releasing and hinge-engaging, we measured hinge-head distance and found the time points that showed stepwise increase or decrease of hinge-head distance by applying step-finding algorithm. At these time points, we measured the hinge-movement angles between the line of two consecutive hinge positions and the line that connecting two heads.

### Dry-AFM sample preparation and imaging

In order to visualize DNA structures, bound by condensin using AFM, we incubated λ-DNA (D1501, Promega) at a concentration of 3 ng/$\ \mu$l with condensin at a concentration of 5 nM in an Eppendorf tube (Fig. 1C) [12, 45]. This mixture was incubated for 10 min to facilitate the interaction between condensin and DNA. Afterward, 1 mM ATP was added, and the sample was further incubated for an additional minute. The resulting solution was then placed on polylysine-treated mica, with a concentration of 0.00001% (wt/vol), for 20 s. The sample-coated mica was rinsed using 3 ml of MilliQ water, followed by drying using N_2_ gas. The AFM measurements were conducted in ambient air using a Bruker Multimode AFM equipped with a Nanoscope V controller and Nanoscope softwar version 9.2 software. Bruker ScanAsyst-Air-HR cantilevers with a nominal stiffness and tip radius of 0.4 N/m and 2 nm were used. The imaging technique employed was PeakForce Tapping, characterized by an 8-kHz oscillation frequency and a peak force setpoint below 100 pN. The angle of condensin anchoring to DNA is defined between the line connecting the hinge and the center of the globular domain of condensin bound to DNA and the DNA tangential direction at the center of the globular domain bound to DNA.

### Model

**Figure 2.**
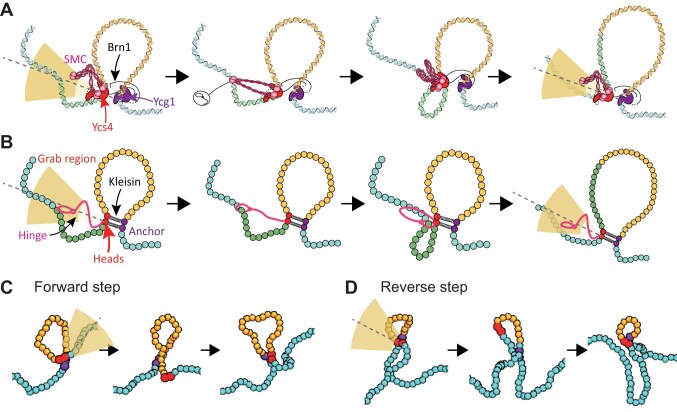
A model for SMC loop formation via anisotropic DNA capture. (**A**) Schematics of the loop extrusion model where the hinge searches for a DNA segment within a truncated spherical sector. The identified segment is then captured and bound to the SMC heads, in turn extending the loop while the hinge returns to the search position. Throughout this process, one DNA segment remains anchored to the Ycg1/Brn1 subunits (purple). (**B**) Implementation of the model on a coarse-grained bead-spring polymer, where the heads and anchor are denoted with red and purple beads, respectively. The hinge is not explicitly modeled with a bead but is accounted for by the geometrically restricted DNA capture region (yellow shaded area). (**C** and**D**) Snapshots from simulations showing loop growth and shrinking as forward (C) and backward (D) steps. Here the anchor is denoted as purple beads while the heads as red beads. The yellow shaded truncated sector indicates the region of segment capture.

We performed MD simulations of an SMC complex operating on linear DNA in the NVT ensemble (Fig. 2 and Supplementary Fig. S2). DNA is modeled as a bead-spring polymer of 400 beads of size $ = 10{\mathrm{\ nm}}$, for a total contour length of 4 $\mu$m. To account for excluded volume interactions, non-bonded beads repel each other according to the Weeks-Chandler Andersen (WCA) potential:


(1) \begin{eqnarray*}
&& {{U}_{WCA}}\left( r \right) = \nonumber\\ && \left\{ {\begin{array}{@{}*{1}{c}@{}} {4\epsilon \left[ {{{{\left( {\frac{\sigma }{r}} \right)}}^{12}} - {{{\left( {\frac{\sigma }{r}} \right)}}^6}} \right] + \epsilon ,\ r \le {{r}_c}}\\ {0,\ r > {{r}_c}} \end{array}} \right.
\end{eqnarray*}

where *r* is the separation between the beads and ${{r}_c} = {{2}^{1/6}}\sigma$. $\epsilon \ = \ {{k}_B}T$ is the energy unit. Neighboring beads are held together by finite-extension-nonlinearelastic (FENE) bonds. Their interaction is governed by the potential:


(2) \begin{eqnarray*}
&& {{U}_{FENE}}\left( r \right) = {{U}_{WCA}}\left( r \right) + \nonumber\\ && \left\{ {\begin{array}{@{}*{1}{c}@{}} { - 0.5kR_0^2\ln (1 - {{{\left( {\frac{r}{{{{R}_0}}}} \right)}}^2}),\ r \le {{R}_0}\ }\\ {\infty ,\ r > {{R}_0}} \end{array}} \right.
\end{eqnarray*}

where $r$ is the distance between the beads, $k\ = \ 30\epsilon /{{\sigma }^2}$ is the spring constant and ${{R}_0} = {\mathrm{\ }}1.5\sigma$ is the maximum extension of the bond. DNA is a semi-flexible polymer with persistence length ${{l}_p}$ ∼ 50 nm (5$\sigma$); we included stiffness in the model by adding a Kratky-Porod energy penalty, acting on triplets of consecutive beads:


(3) \begin{eqnarray*}
{{U}_{KP}}\left( \theta \right) = \frac{{{{k}_B}T{{l}_p}}}{\sigma }\left[ {1 - \cos \left( \theta \right)} \right]
\end{eqnarray*}

where $\theta$ is the angle formed by the three beads of a given triplet.

Each SMC protein is modeled as a pair of springs bringing together two short DNA segments made of pairs of neighboring beads (Fig. 2B and Supplementary Fig. S2A). This is practically achieved by using two harmonic bonds, described by the potential:


(4) \begin{eqnarray*}
{{U}_{KP}}\left( \theta \right) = k{{\left( {r - {{r}_0}} \right)}^2}
\end{eqnarray*}

where $r$ is the distance between the bonded beads, ${{r}_0}\ = \ 1.6\sigma$ is the resting distance and $k\ = \ 5\ \epsilon /{{\sigma }^2}$ is the elastic constant. We chose this constant to be small on purpose, to mimic the weak constraint imposed by the kleisin subunit. To integrate the equations of motion we used the LAMMPS package [46]. The integration timestep is set to $dt\ = \ 0.01{{\tau }_B}$, where ${{\tau }_B}$ is the Brownian time of a DNA bead. All simulations are performed in at least 30 independent replicates to obtain statistically significant averages and standard deviations.

To load an SMC on DNA, first we randomly selected two DNA beads, which must be proximate in 3D (distance < 4$\sigma$), representing the position along DNA of the anchor and the head of the SMC. Then, the two harmonic bonds modeling the SMC are added: one connects the head to the anchor, the other connects the two beads upstream (i.e. inside the extruded loop, Supplementary Fig. S2A). Note that to do this, the two randomly chosen beads must be at least 2 beads apart. To practically simplify the addition and removal of the harmonic bonds, we required them to be at least 3 beads apart.

Once loaded, we attempted to update the position of the springs at regular intervals $\Delta t$. The update rule for the extrusion follows these rules: suppose that a SMC loaded at time ${{t}_0}$ attempts to take a step at time $t = {{t}_0} + n\Delta t, n \in \mathbb{N}$, and let us call ${{r}_h}( t )$ and ${{r}_a}( t )$ the position of the head and anchor at time $t$, and ${\mathrm{dir}}( t )\ = \ {{r}_h}( t )\ - \ {{r}_a}( t )$ the vector going from the anchor to the head. First, all DNA beads for which the position at time $t$,$\ r( t )$, satisfies


(5) \begin{eqnarray*}
\frac{{{\mathrm{dir}}\left( t \right) \cdot {\mathrm{pos}}\left( t \right)\ }}{{\left| {{\mathrm{dir}}\left( t \right)} \right|\left| {{\mathrm{pos}}\left( t \right)} \right|}} \le \cos \left( {\gamma /2} \right)
\end{eqnarray*}

where ${\mathrm{pos}}( t )\ = \ r( t )\ - \ {{r}_h}( t )$ and $\gamma$ is the grabbing angle, and $\sigma \ \le \ | {{\mathrm{pos}}( t )} |\ \le \ 4\sigma$, are identified. Then one of these beads (if any) is randomly selected (Supplementary Fig. S2A) and the current bead corresponding to the heads position is deleted and two new bonds, connecting the anchor to the new head, and their upstream neighbors are added to the DNA. We highlight that this procedure extracts work by manually stretching the spring associated with the heads and allowing it to return to its equilibrium position. We do this to implicitly mimicking the consumption of ATP, known to change conformation in SMCs such as condensin.

If none of the DNA beads at a given timestep satisfy the conditions above, the bonds are left unchanged. Note that, whereas the head can be updated at every step, the anchor never changes. Additionally, we do not impose direction *a priori*, and these selection rules can choose any bead up- or downstream of the current position of the head mimicking random 5′→3′ or 3′→5′ directions. For simplicity, to follow the growth of the extruded loops without disruptions, we also disallow big jumps in extruded length by excluding from the selection rule beads which are farther than 5 beads in 1D (∼150 bp) from the current head position; however, our results are unchanged if we extend this limit to 20 beads (∼600 bp). In other words, we set what we previously defined inter-strand grabbing probability to 0 [36]. We fix $\Delta t\ = \ {{10}^2}{{\tau }_B}$ (equivalent to 0.023 s) and run each simulation for ${{10}^5}{{\tau }_B}$ (∼23 s).

To check whether and how the update time and the stiffness of the bonding springs affect the extrusion dynamics, we ran simulations with different values of $k$ and $\Delta t$. The average extrusion lengths predicted for $k\ = \ 2,\ 5$ and $8\ \epsilon /{{\sigma }^2}$ are similar to each other, showing that the spring stiffness does not affect the result (Supplementary Fig. S2B). Supplementary Fig. S2C verifies that the value of $\Delta t$ we chose is large enough to allow the local equilibration of DNA, and that, for large enough $\Delta t$, regardless of its value, the extrusion speed is a function of the number of attempted steps.

## Results

### AFM reveals that yeast condensin hinge extends within a truncated circular sector orthogonally to the bound DNA.

We hypothesized that the hinge motion presented some geometric restrictions due to its connection to structured coiled-coil arms [35]. To test this, we performed and analyzed high-speed (HS)-AFM imaging of yeast condensin in liquid at a temporal resolution of 200 ms to determine the typical position of the hinge relative to the globular head domains [12] (Fig. 1, Supplementary Fig. S1, and Supplementary Movie). We could identify two distinct globular domains: the hinge and the heads, linked together by semi-flexible arms (Fig. 1A). We could also distinguish the hinge-released and the hinge-engaged states by measuring the distance of the hinge from the heads (Supplementary Fig. S1B and D, and Supplementary Video S1). Thus, we measured the angles of hinge-released and hinge-engaged states, from the midpoint of the two heads, with respect to the line connecting two heads (Fig. 1A and B) and discovered that the hinge is often extended orthogonally to the line joining the SMC heads (Fig. 1A and B). More specifically, the angles are normally distributed and peaked around $90^\circ$ for both hinge-released (mean ± s.d. = 83 ± 29°) and -engaged (mean ± s.d. = 86 ± 44°) states.

To validate our findings, we performed dry-AFM imaging of DNA-bound condensin and measured hinge extension angles (Fig. 1C and Supplementary Fig. S1E). While liquid-phase HS-AFM is well-suited for probing DNA–condensin dynamics, its limited temporal resolution made it difficult to clearly visualize hinge motion, as the hinge region could not be reliably distinguished from the DNA. Thus, we consider liquid-phase imaging a future objective. In contrast, dry AFM offers the advantage of imaging large surface areas, enabling structural analysis over tens of micrometers. Accordingly, we clarified the complementary roles of each technique: HS-AFM is used to observe hinge dynamics (Fig. 1A and B, and Supplementary Fig. S1A–D), while dry AFM provides statistically robust structural data (Fig. 1C and Supplementary Fig. S1E). The dry-AFM measurements confirmed near-orthogonal hinge extension, with a Gaussian distribution centered at *α* = 88 ± 23° (mean ± s.d.), and the distribution width defines an angular range *γ* = 2 × 23° = 46°, representing the circular sector explored by the hinge (Fig. 1C and D). To account for the broader distributions observed in liquid-phase HS-AFM, we used *γ* = 60° as a representative value unless otherwise noted (Fig. 1A and B). Additionally, we obtained the distribution of hinge–head distances, capturing the transition between hinge-released (∼40 nm) and hinge-engaged (∼10 nm) states (Supplementary Fig. S1D) [12, 28].

Finally, our measurements indicated that the hinge covers a “truncated circular sector” that extends orthogonally to the bound DNA, with $\alpha \simeq 90^\circ ,{\mathrm{\ }}\gamma \simeq 60^\circ ,{\mathrm{\ }}$and a reach spanning 10–40 nm from the DNA (Fig. 1D). In 3D, the hinge motion defines a “truncated open spherical sector”, created by the 2π rotation of the circular sector around the DNA. Although our AFM measurements were performed in 2D, this geometry suggests that in 3D, the hinge covers a volume corresponding to the solid of revolution formed by this rotation. We defined this hinge-reachable region for the scrunching model and explored how variations in *γ* influence the loop extrusion process.

### A SMC model with anisotropic DNA capture in 3D

Motivated by our experiments, we propose a new geometrically constrained “DNA capture” or “DNA bridging” model to explain the formation of loops by SMCs, specifically yeast condensin. In this model, the Ycg1/Brn1 subunits bind DNA irreversibly via a “safety belt” mechanism and thus act as “anchors” [47], while the SMC heads/Ycs4 bind another section of DNA reversibly [22] (Fig. 2A). We then assume that the hinge can capture (or bind) a new DNA segment within the truncated circular sector that we found above: direction $\alpha = 90^\circ$ and width $\gamma$ from the bound DNA (Fig. 1D). Then, ATP binding induces a conformational change that brings the captured DNA close to the heads/Ycs4 subunits. Finally, ATP-hydrolysis induces the heads/Ycs4 re-bind to the newly captured DNA segment—thereby extending (or shrinking) the extruded loop—and the hinge is then free to target a new DNA segment (Fig. 2A). These steps follow an irreversible cycle through protein states and thus require ATP consumption to violate detailed balance (i.e. violate time reversal symmetry). However, there is no guarantee that this irreversible cycle leads to loop growth, as the larger the loop, the more likely it is for the SMC to make a “mistake” and capture a DNA segment inside the loop, thus leading to a backward step, i.e. a loop shrinkage.

To simulate this model, we implemented a coarse-grained loop capture process with a geometric constraint on the region that can be reached by the hinge. Specifically, we account for the connectivity of the anchor (Ycg1) to the heads (Smc2 and Smc4) via the kleisin subunit (Brn1) as beads connected by relatively weak ($5{{k}_B}T/{{\sigma }^2}$) harmonic bonds, implying that the heads and anchor segments move with respect to each other (Fig. 2B and D). Additionally, we impose that the search of the DNA segment to capture is to be performed within an open spherical sector (3D version of a 2D truncated circular sector) extending orthogonally to the SMC-bound DNA (Fig. 2B). When a segment of the coarse-grained polymer falls within the open spherical sector and within a certain Euclidean distance (10 and 40 nm) the position of the harmonic bond connecting anchor and heads is then stretched to grab such new segment (Fig. 2B): the harmonic spring connecting anchor and heads is temporarily extended and allowed to return to its equilibrium distance, in turn bringing the captured segment close to the anchor. Finally, the new DNA segment is identified with the new position of the heads, the anchor remains at its original position, and the hinge is then returned free to search and capture a new DNA segment within the truncated open spherical sector (Fig. 2B). This model covers a full ATP-cycle and involves a 3D search of proximal DNA segments where the geometric constraint is defined by the opening angle $\gamma$. The smaller the value of $\gamma$, the smaller the region within which the hinge can capture a new DNA segment. Note also that this cycle breaks detailed balance as it is not reversible (captured segments are never pushed away from the anchor but only pulled closer). At the same time, it is important to realize that this broken detailed balance (broken time reversal symmetry) does not guarantee systematic loop extension, as backward steps are still allowed within the model and are expected to become significant as the loop becomes longer (Fig. 2C and D, and Supplementary Fig. S2). As we shall see below, systematic loop growth requires both broken detailed balance and anisotropic DNA capturing.

The important difference from previous models of loop extrusion [1–3, 25, 36, 48, 49] is that we do not impose an *a priori* bias on the growth of the loop. The hinge can capture any segment ahead, or behind, the current 1D position of the heads. In fact, in our simulations, we observed backward extrusion steps, where the newly grabbed segment is inside the extruded loop, thus reducing the loop length (Fig. 2D). Importantly, this is in line with experiments, where backwards steps are also often observed [33].

Our model also accounts for an additional spring to mimic the presence of the disordered kleisins attaching the anchor Ycg1 to the SMC heads. This additional spring imposes a weak constraint on the relative rotation of the heads-bound and anchor-bound DNA segments to avoid that these segments fully twist around each other by >90°. This effectively mimics the presence of a flexibly linked protein structure (kleisin, Brn1 in yeast condensin) that maintains a weak alignment [22].

### Anisotropic 3D capture is necessary to yield rectified loop extrusion

**Figure 3.**
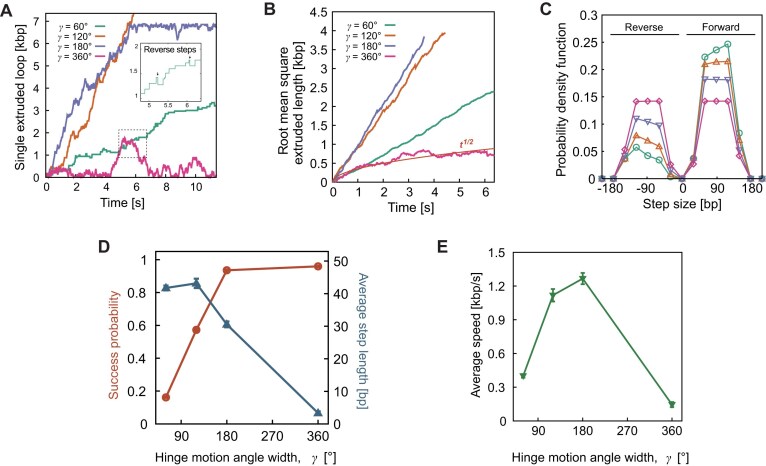
Self-rectified loop extrusion emerges from anisotropic capturing of DNA segments. (**A**) Individual traces of simulated extruded loops as a function of time and with different hinge-search angles $\gamma$. The inset shows a zoomed-in view of the trace at *γ* = 60°, which occasionally exhibits backward steps. (**B**) Root mean squared extruded length as a function of time and for different hinge-search angles. The isotropic case ($\gamma = {\mathrm{\ }}360^\circ$) yields a random walk scaling as ${{t}^{1/2}}$. (**C**) Probability of step size as a function of the search angle: the narrower the angle, the larger the forward/reverse ratio and the more rectified the extrusion. (**D**) Plot of the success probability and average step sizes as a function of the hinge search angle. (**E**) The trade-off yields an optimum of the extrusion velocity at $\gamma = 180^\circ$.

Using this model, we simulated loop formation on a bead-spring polymer with $N$ = 400 beads of size $\sigma$ = 10 nm ($ \simeq$12 kbp), and with varying opening angles $\gamma$. We then tracked the positions of the anchor and heads and defined an oriented loop length as $l\ = \ {{n}_a}\ - {{n}_h}$, where ${{n}_a}$ and ${{n}_h}$ are the positions of the anchor and the heads, respectively. For $\gamma < {\mathrm{\ }}360^\circ$, the SMCs displayed persistently growing loops with a clear sign of rectification (Fig. 3A and Supplementary Fig. S2). Strikingly, the directed loop growth occurs until the loop size is comparable to the length of the polymer. Even at large times, when the extruded loop has a similar, if not longer, length of the outside tails of the chain, the SMC continues to grow, rather than shrink, the loop (Supplementary Fig. S3). This observation suggests that different dynamics of inner loop and outer tails do not contribute to the rectification and is supported by the fact that this behavior is not affected by the stepping rate. This implies that the stepping rate is slow enough to allow full equilibration of the polymer segment inside and outside the formed loop. This is likely the case also in experiments, as SMC ATP hydrolysis rate is slow (∼2 ${\rm molecules}/{{s}^{ - 1}}$) [10, 11].

Importantly, and as expected, in the spherically symmetric case ($\gamma = {\mathrm{\ }}360^\circ$), we do not observe rectified loop growth. Instead, the loops formed in between the anchor and heads tend to shrink back to zero. They effectively behave as random walks with a reflecting wall positioned at the anchor (Fig. 3A). This is important because it indicates that ATP hydrolysis and work done by pulling DNA into the complex is not enough to systematically grow (or extrude) a DNA loop; instead, a DNA capture model requires an anisotropic search in order to explain unidirectional loop growth and absence of significant backward steps.

**Figure 4.**
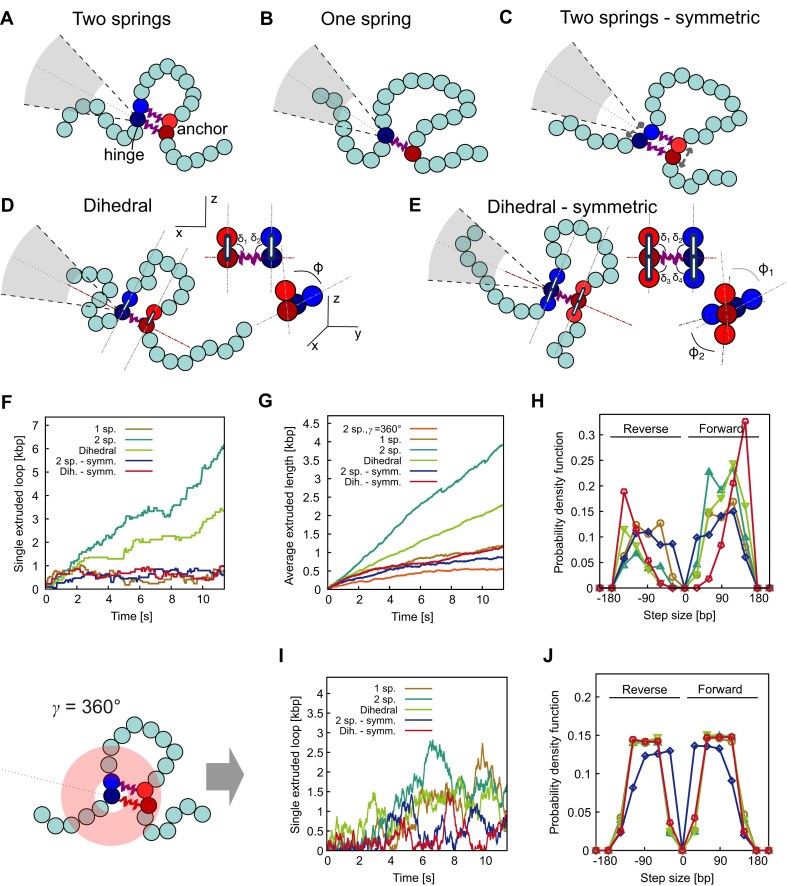
Effect of different geometric local constraints on DNA capture. (**A–E**) Sketch of different models to simulate local DNA geometric constraints imposed by SMC binding. (**F** and**G**) Comparison of individual (F) and average (G) extruded length as a function of time as predicted by the models depicted in panels (A–E). The orange line shows the case with $\gamma$ = 360° representing a pure random walk. (**H**) Step size distributions. (**I** and**J**) Individual extruded length (I) and step size distributions (J) predicted by the models sketched in panels (A–D) but with $\gamma$ = 360° instead of $\gamma$ = 60°.

To further characterize this process we took the root mean squared extruded length $\langle l\rangle \ = \ {{\langle {{{{[ {{{n}_a} - \ {{n}_h}} ]}}^2}} \rangle }^{1/2}}$ and indeed found that the spherically symmetric case displayed a scaling $\langle l\rangle \ \sim \ {{t}^{1/2}}$, as a simple random walk (Figs 3B, and 4I and J). Interestingly, we also noted that the cases with $60^\circ < {\mathrm{\ }}\gamma < {\mathrm{\ }}360^\circ$ displayed faster linear growth than the case with $\gamma = {\mathrm{\ }}60^\circ$. Despite this, the distribution of step sizes clearly indicates that $\gamma = {\mathrm{\ }}60^\circ {\mathrm{\ }}$is the one that benefits from the greater rectification, i.e. the ratio forward/reverse steps are the largest (Fig. 3C). In general, wider angles increased the probability of shorter or backward steps, while narrow ones favored longer, forward steps (Fig. 3C). In turn, the average step size—defined as $s\ = \ {{\Sigma }_i}[ {sign( i ){{S}_i}} ]/N$, where ${{S}_i}$ is the $i$-th step size—was typically smaller for wider angles. The largest probability of large steps ∼50 nm, in line with that seen in experiments [29], was obtained for $\gamma = {\mathrm{\ }}60^\circ$, close to the one measured in our AFM experiments (Fig. 1).

To understand why wider capture angles yielded faster extrusion in our simulations, we computed the probability of successful stepping. Since we impose that SMCs do not make a step if, in a given simulation time, there are no DNA beads that satisfy the search criterion, we expect (and observe) that narrower search angles naturally yielded lower successful stepping probability (Fig. 3D). The opposite trends of successful stepping probability (increasing with $\gamma$) and average step size (decreasing with $\gamma$) yields a trade-off that naturally leads to an optimum in velocity around $\gamma \simeq 180^\circ$ (Fig. 3E), which is somewhat larger than the experimentally observed $\gamma \simeq 60^\circ$ seen above.

Interestingly, we repeated our simulations in quasi-2D by confining the polymer within a narrow slab and discovered that the optimal grabbing angle shifted to narrower values, in the range $\gamma \simeq 60^\circ - 120^\circ$ (Supplementary Fig. S4). This suggests that the deposition of the protein complex on the surface prior to AFM imaging may be affecting the region sampled by the hinge and that, if we could measure the complex in 3D, the hinge would span a wider angle (see Supplementary Information).

By restricting the DNA capture angle, the probability of performing a successful step is also reduced. For this reason, although the case ${\mathrm{\gamma }} = {\mathrm{\ }}60^\circ$ maximizes the forward/backward steps ratio, it also displays the highest rate of unsuccessful captures, thus reducing the overall loop growth.

We highlight that since yeast condensin can make large steps [33], it is thus not necessary that all ATP cycles are converted into steps to reach speeds observed *in vitro*. Even for one of the most precise angles of 120 degrees, we still have ∼60% success rate of capturing a DNA segment. Considering that at every successful grab condensin can reel in up to 200 bp or more [33], and that each SMC complex makes a few ATP cycles per second [10, 45], then our model is compatible with a speed of 0.1–1 kbp/s [10]. Additionally, as we show in the next section, our model predicts faster loop growth on tethered DNA and is thus even more in line with experiments.

We conclude this section with a further interpretation of this key result. Our simulations suggest that the intrinsic SMC structure impose a physical constraint on the DNA capture step; in other words, the hinge has a limited “visibility” of the available DNA around the SMC complex. This constraint makes it more likely that DNA segments outside the extruding loop are being captured. The physical picture that emerges is that, structurally, the SMC complex is “facing the growing loop” and the hinge is “capturing segments outside its field of view” thus ensuring that the complex is not capturing segments within the same loop. Our simulations suggest that this structural constraint, coupled with an ATP-consuming process that generates work, is sufficient to generate a rectified loop extrusion process with negligible backward steps.

### Anisotropic capture on tethered DNA

To more closely mimic single-molecule *in vitro* experiments, we have also explored how pulling the DNA ends with a constant force affects the extrusion directionality. This set up is akin to that of DNA tethered to a surface or stretched with optical or magnetic tweezers and we have already demonstrated that our model of loop extrusion *in trans* is able to recapitulate the observations of loop extrusion *in vitro* [33, 36]. Here, we demonstrate (see Supplementary Fig. S5) that increasing the stretching force enhances the rectification of the loop growth process, with the SMC taking even fewer reverse steps compared with the non-tethered case. This effect arises from the difference in tension between the DNA segments outside and inside the extruded loop. Stretching the DNA ends effectively strengthens the local conformational asymmetry near the boundaries of the extruded loop, thereby promoting the SMC’s ability to maintain its directional movement. Our observations thus suggest that loop extrusion performed on tethered or stretched DNA substrates such as in [7, 8, 10] is more efficient than on relaxed DNA, simply because of the reduced entropy in the tethered assay. This finding also suggests that: (i) even if an SMC performed “loop capture” in 3D space, it may effectively appear to perform loop extrusion *in cis* (in 1D) when constrained to act on a tethered and/or stretched DNA molecule (Supplementary Fig. S6) and (ii) the measurements performed on tethered loop extrusion may yield larger efficiencies than the one *in vivo*.

### Effects of asymmetric DNA binding on rectification

To investigate the other factors contributing to the rectification, we simulated different models, including asymmetric capture and torsional constraint. The model described previously (Fig. 2) incorporates two springs and a capture region positioned such that the beads within the extruded loop—those next to the anchor, $a$, and head, $h$—are closer than those outside the loop. We chose to impose this structural asymmetry with weak springs ($k\ = \ 5\ \epsilon /{{\sigma }^2}$) reflecting the intrinsically disordered nature of the kleisin subunit (e.g. Brn1 in yeast condensin). Additionally, the bonded beads representing segments near the head and anchor are treated as “flexibly linked” [2] allowing for rearrangement. In this model, the hinge-mediated search area axis passes through $a$ and $h$, and so it contributes to creating a local asymmetry that favours the capturing of DNA segments outside the loop and in turn promoting loop growth (Fig. 4A and Supplementary Fig. S7A).

To test this hypothesis, we restored this local symmetry either by (i) using one spring instead of two springs (Fig. 4B and Supplementary Fig. S7B) or (ii) by repositioning the capture region such that its axis passed through the barycentre of the bonded beads (Fig. 4C and Supplementary Fig. S7C). In Fig. 4F–J, we show that rectification was significantly more efficient in the asymmetric configuration. These results demonstrate that a structural asymmetry near the SMC’s DNA binding sites may enhance rectification and loop growth.

### Effects of torsional constraint on rectification

Motivated by the structural chirality between the two bound DNA regions observed in yeast condensin cryo-EM [22], we studied the effect of introducing torsional constraints between the two bound DNA segments on loop capture and rectification. This was simulated by replacing one of the two springs with an angular energy penalty applied to the dihedral angles formed by the bonded DNA segments (as shown in Fig. 4E and Supplementary Fig. S7E). The asymmetric potential was defined as:


(6) \begin{eqnarray*}
{{U}_{asymm}}\ = \ {{U}_{KP}}\left( {{{\delta }_1}} \right) + {{U}_{KP}}\left( {{{\delta }_2}} \right) + {{U}_{quad}}\left( {{{\varphi }_1}} \right)
\end{eqnarray*}

where ${{\delta }_1}$, ${{\delta }_2}$, and ${{\varphi }_1}$ are the angles depicted in Fig. 4D and Supplementary Fig. S7D. ${{U}_{KP}}$ represents the Kratky-Porod angle potential described in Eq. 3 (see “Materials and methods” section), and ${{U}_{quad}}( \varphi )$ is defined as:


(7) \begin{eqnarray*}
{{U}_{quad}}\left( \varphi \right)\ = \ 5\epsilon {{\varphi }^2}
\end{eqnarray*}

Note that the model in Fig. 4D has only one spring that connects those two outermost beads in the loop. The symmetric version of this model is instead illustrated in Fig. 4E and Supplementary Fig. S7E, where given by the following potential:


(8) \begin{eqnarray*}
&& {{U}_{symm}} = {{U}_{KP}}\left( {{{\delta }_1}} \right) + {{U}_{KP}}\left( {{{\delta }_2}} \right) + {{U}_{KP}}\left( {{{\delta }_3}} \right) + {{U}_{KP}}\left( {{{\delta }_4}} \right) + \nonumber\\ && {{U}_{quad}}\left( {{{\varphi }_1}} \right) + {{U}_{quad}}\left( {{{\varphi }_2}} \right)
\end{eqnarray*}

where ${{\delta }_1}$, ${{\delta }_2}$, ${{\delta }_3}$, ${{\delta }_4}$, ${{\varphi }_1}$, and ${{\varphi }_2}$. The symmetric potential (${{U}_{symm}}$) favors conformations where all four segments next the boundaries are orthogonal to the axis of the capture region.

Simulations show that loop rectification occurred only in the asymmetric dihedral model, not in the symmetric torsional constraint model. This suggests that torsional constraints are not critical for rectified loop extrusion, though their inclusion increased the forward step size (Fig. 4H). The extruded length and step distributions with $\gamma = {\mathrm{\ }}60^\circ {\mathrm{\ }}$and 360° under all the configurations that we have simulated, presented in Fig. 4F and G. Strikingly, the results resembled those observed with the original spring-based model, shown in Fig. 2B. Under ${{U}_{asymm}}$, rectified motion was evident (Fig. 4F and G, “dihedral”), whereas no rectification was observed for ${{U}_{symm}}$ (Fig. 4F and G, “dihedral—symm”). This was further corroborated by the step-size distributions: with asymmetric under ${{U}_{asymm}}$ and symmetric under ${{U}_{symm}}$.

Importantly, regardless of the dihedral potential, an isotropic grabbing angle ($\gamma = {\mathrm{\ }}360^\circ$) resulted in random walks without rectification (Fig. 4I and J). These results demonstrate that rectified loop extrusion requires anisotropic DNA loop capture, and that local structural asymmetry in the DNA binding enhances the rectification.

## Discussion

In this study, we investigated a previously overlooked mechanism behind DNA loop formation driven by SMC complexes, focusing on the processes maintaining rectified DNA loop growth. Using dry and liquid HS AFM, we observed that the hinge domain of yeast condensin exhibits anisotropic motion orthogonal to the bound DNA while extending to capture new DNA segments. This geometric constraint, coupled with the intrinsic broken detailed balance and the asymmetric arrangement of DNA binding to heads and non-SMC subunits, leads to rectified loop extrusion. Indeed, a SMC model with isotropic DNA capture yields both loop growth and shrinkage, thus behaving as a random walk. We thus argue that anisotropic DNA capture by the hinge is critical for rectified DNA-loop extrusion and is directly encoded in the structure of SMC complexes.

### Determinants for rectified loop extrusion

We have provided experimental evidence from dry and liquid HS AFM that the structure of yeast condensin favors certain geometric conformations where the hinge is extended orthogonally to the local direction of the heads-bound DNA segment and within an open circular sector subtended by an angle $\gamma \in [ {40^\circ {\mathrm{\ }}:{\mathrm{\ }}60^\circ } ]$ (Fig. 1), in turn rendering the search process anisotropic. The observed angular restriction of the hinge movement, within a well-defined circular sector, limits the regions of DNA that the hinge can search. This bias ensures that the complex preferentially grabs DNA outside of the extruded loop, promoting forward extrusion while minimizing the likelihood of backward steps (Fig. 3). Interestingly, our simulations reveal a trade-off between search efficiency and step size, driven by the hinge’s search angle. We find that the narrower the angle *γ*, the larger the typical step size and the more rectified the extrusion, but also the more likely to fail to find a DNA segment to grab in a given time. This trade-off results in an optimal extrusion speed at an intermediate search angle (∼180°), but the experimentally observed angle (∼60°) suggests that condensin operates within a more constrained search space, potentially to optimize the search process in the crowded chromosomal environment *in vivo*. We should also note that our simulations suggest that in 2D the optimal DNA capture angle may be smaller (see Supplementary Fig. S4), in line with our AFM data. When the hinge search is allowed to span an isotropic, spherical region around the SMC complex, the model produces loop extrusion dynamics similar to a random walk. This finding underscores the critical role of constrained hinge motion in driving loop growth over shrinkage, and we argue that this constraint emerges naturally from the structure of SMC complexes.

We propose that the emergence of spontaneously rectified loop extrusion results from a combination of broken detailed balance and geometric structural constraints on the hinge-mediated 3D search. In this study, we demonstrate that for SMC complexes, the combination of (i) an anisotropic hinge motion (as in the scrunching model) and (ii) a structural constraint during the DNA-grabbing step is enough to explain the rectified loop extrusion observed in experiments. This mechanism differs from previously proposed models that rely on molecular motors or active chiral elements to enforce directionality. For example, the directionality of kinesin and myosin is driven by ATP-fueled movement along polarized substrates, such as microtubules or actin filaments [50, 51]. In contrast, SMC complexes act on non-polarized DNA molecules. Here, rectification arises passively from the intrinsic geometry of the protein–DNA complex and the energy input from ATP hydrolysis, which breaks detailed balance.

### Structural implications

Recent structural studies indicate that both cohesin and condensin complexes feature asymmetrically oriented HEAT-repeat subunits (Ycg1 and Ycs4 in yeast condensin; Scc2 and Scc3 in yeast cohesin) [22, 52, 53]. The two HEAT-repeat subunits display DNA-binding affinities, with DNA segments dangling within the kleisin IDR region. This region asymmetrically bridges the two SMC proteins by linking one DNA segment to the head of one SMC and the other to the neck of the second SMC [19]. Furthermore, structural studies have demonstrated ATP-dependent asymmetric dimerization of the heads, suggesting a subtle bias in the interaction between heads and DNA segments [20, 22]. These structural asymmetries may impose asymmetric DNA engagement at the heads/non-SMC subunits, leading to a constraint in the DNA capture region sampled by the hinge and, as we show in this paper, in turn promoting rectified DNA loop extrusion. We argue that future structural and biophysical studies on the spatiotemporal dynamics of SMC complexes during loop formation will be critical to clarify the underlying mechanism.

### Comparison with other models

Our AFM images suggest that SMCs folding the coiled-coli arms in an open configuration, consistent with the scrunching model [12, 25, 28, 33]; however, our simulations do not exclude alternative mechanisms, such as the DNA-segment capture model or the reel-and-seal model. In the reel-and-seal model, the hinge encloses a looped DNA segment, which is subsequently reeled in as part of the loop extrusion process [11, 41]. Similarly, in the DNA-segment capture model, the hinge transiently captures a new DNA segment and transfers it to the heads for extrusion [34]. A shared feature across these models is the hinge’s role in targeting a new DNA segment. The key message of our work is that, due to geometric constrains, the hinge samples an anisotropic region during the DNA capture step and this anisotropy allows loops to grow systematically, rather than grow and shrink back. Moreover, our model naturally explains backward steps observed in magnetic tweezers experiments, whilst predicting an overall forward motion. Indeed, our simulations can align with all three models, providing a plausible explanation for the origin of rectified loop extrusion. Further investigations into the structural dynamics of SMC complexes and their interactions with DNA during loop extrusion will be crucial for refining our understanding the role of the hinge.

In conclusion, anisotropic search mechanisms and ATP-driven disruption of detailed balance together can explain the spontaneous rectified loop extrusion by SMC complexes. Due to the shared structural features between yeast condensin, cohesin, and bacterial condensin, we argue that this self-rectifying loop extrusion mechanism may be conserved across different SMC complexes. Our work opens several new avenues for future research. For example, protein-engineering experiments that alter the flexibility of the SMC coiled-coil arms or making symmetric arrangements of heads and non-SMC subunits could directly test the importance of the hinge’s angular constraint in loop extrusion. Similarly, modifying the DNA environment *in vitro* could further elucidate the role of crowding and other biophysical factors in SMC-driven genome organization. Furthermore, it will be of interest to explore whether similar constraints are at play in other ATP-dependent chromatin remodelers or DNA-binding proteins, extending the relevance of our findings to a broader class of molecular machines involved in genome maintenance.

## Supplementary Material

gkaf725_Supplemental_Files

## Data Availability

The data underlying this article are available in the article and in its online supplementary material.
