# Supplementary material for: Spontaneously directed loop extrusion in SMC complexes emerges from broken detailed balance and anisotropic DNA search
Source: Nucleic Acids Res. 2025 Aug 1;53(14):gkaf725. doi: 10.1093/nar/gkaf725 (PMC12313344; doi:10.1093/nar/gkaf725)
Supplement: gkaf725_Supplemental_Files [file gkaf725_supplemental_files.zip › NAR_versions SMC article_supple_v3.pdf]

Supplementary Information

Spontaneously Directed Loop Extrusion in SMC complexes Emerges from Broken Detailed Balance and Anisotropic DNA Search

Andrea Bonato[1,*], Jae-Won Jang[2], Do-Gyun Kim[3], Kyoung-Wook Moon[3], Davide Michieletto[4,5,*] and Je-Kyung Ryu[2,3,*]

[1] *Department of Physics, University of Strathclyde, Glasgow, G4 0NG, UK*

[2] *Interdisciplinary Program in Computational Science, Seoul National University, Seoul, 08826, South Korea*

[3] *Department of Physics and Astronomy, and Institute of Applied Physics, Seoul National University, Seoul, 08826, South Korea*

[4] *School of Physics and Astronomy, University of Edinburgh, Peter Guthrie Tait Road, Edinburgh, EH9 3FD, UK*

[5] *MRC Human Genetics Unit, Institute of Genetics and Cancer, University of Edinburgh, Edinburgh EH4 2XU, UK*

[*] Correspondence: andrea.bonato@strath.ac.uk, Davide.Michieletto@ed.ac.uk and prof.love@snu.ac.kr

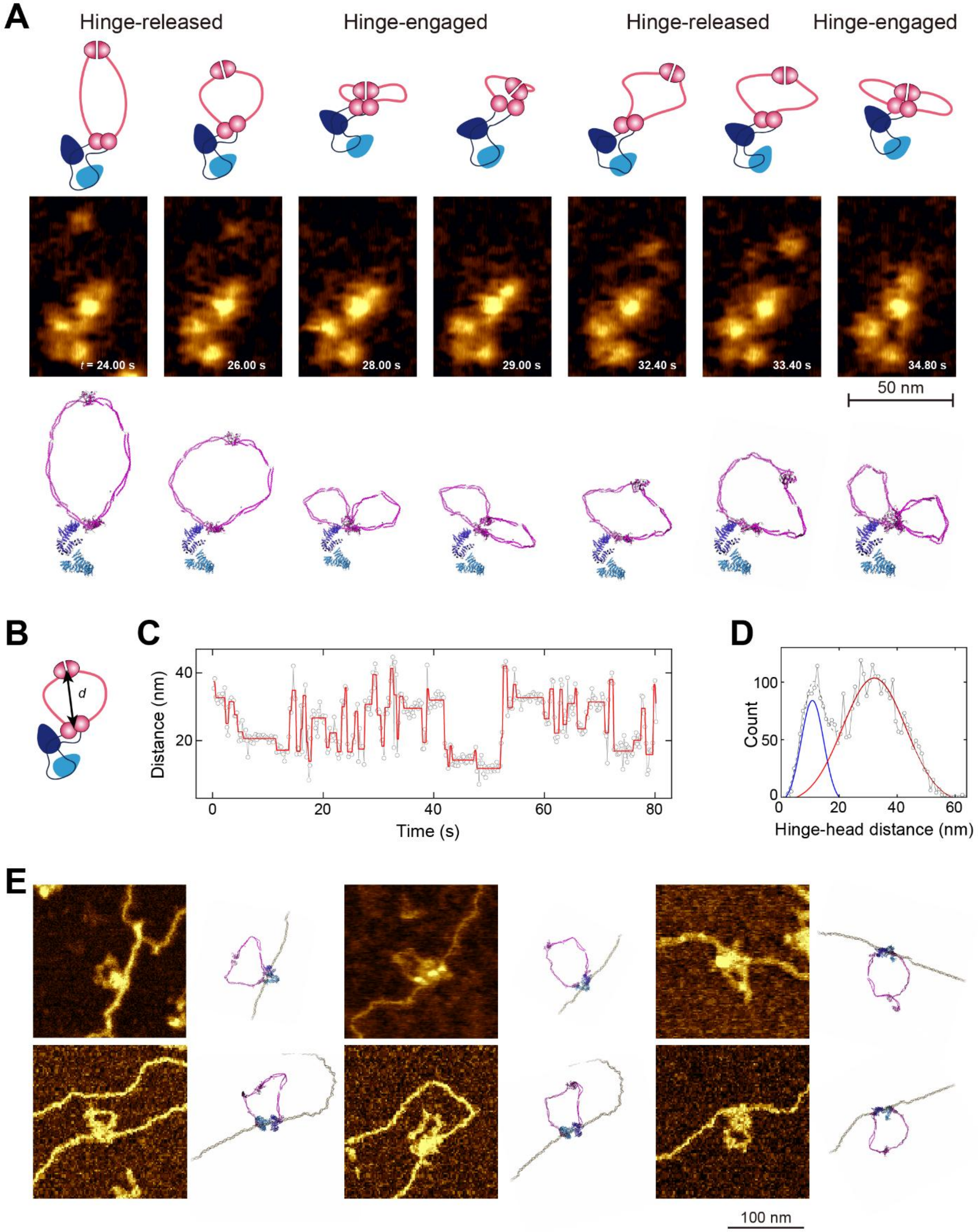

Supplementary Figure 1. AFM analysis of yeast condensin holocomplex. **(A)** Liquid high-speed AFM images of yeast condensin extending and retracting the hinge (notice the position of the small globular

structure at the top/top-right of the images, *t* = 26.0, 29.0, 32.0, 36.6., 37.6, 38.2, and 38.4 s). Cartoon models of yeast condensin (top), HS AFM images (middle), and PDB structure models (PDB number: 6YVU (1,2), bottom). **(B–D)** Real-time traces of hinge-motion of yeast condensin holocomplex. **(B)** Cartoon of condensin holocomplex. Hinge-head distance was measured for every HS AFM frame. **(C)** Real-time traces of hinge-head distance changes. To measure the angles of hinge movement respect to the head-head line, the vector constructed by the hinge movement at two consecutive frames where stepwise hinge motion is observed. To find the timepoint when stepwise hinge motion is observed, we applied an automatic step-finding algorithm previously developed in Ref. (3). **(D)** Two gaussian distributions of hinge-head distances (*N* = 3,484 frames, mean ± SD = 10.8 ± 3.4 and 32.1 ± 11 nm). These distributions define the hinge-reachable truncated-cone shaped region. **(E)** More dry-AFM images of DNA-bound condensin (left) and PDB structure models (PDB number: 6YVU (1,2), right) (*N* = 79).

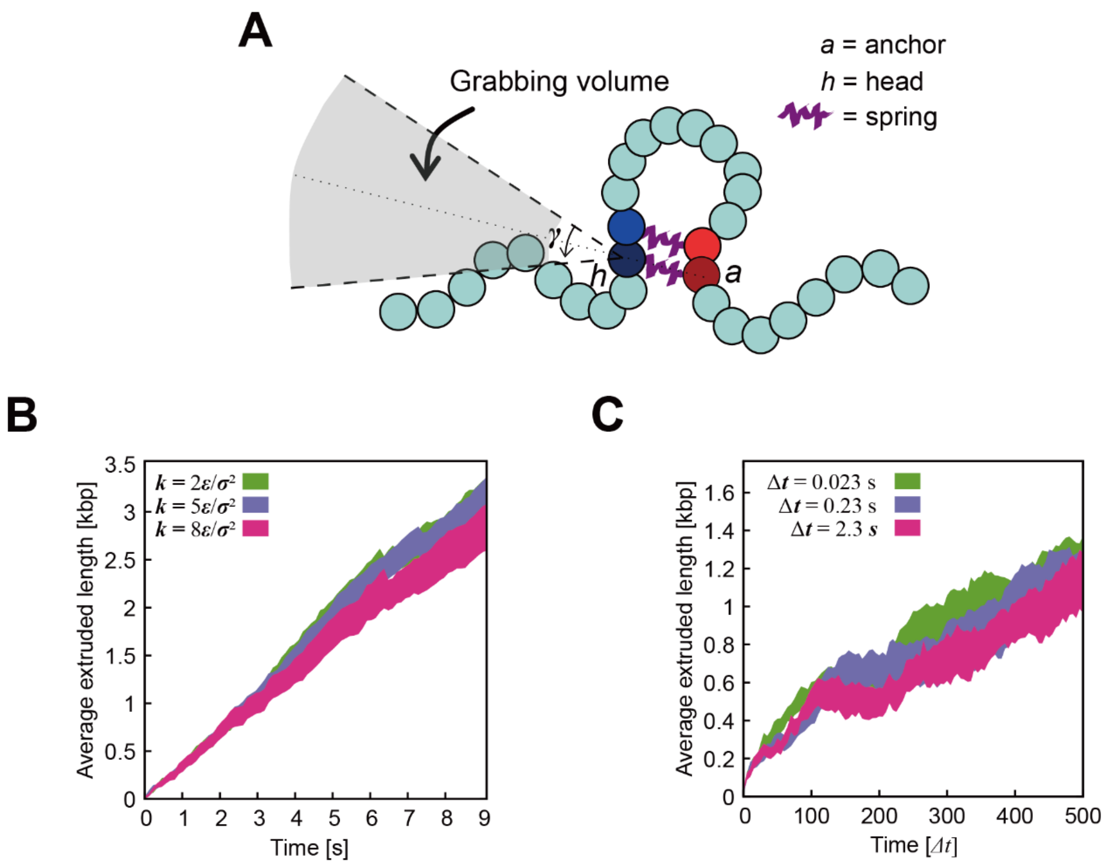

Supplementary Figure 2. Model sketch and dependency on spring stiffness and stepping time. **(A)** Sketch of the loop extrusion model. The LEF is modelled as two harmonic springs holding together a DNA loop and the position of the head, at the base of the loop, is updated (i.e. a new head is selected) every Δt timesteps. Candidate capture beads are searched within the capture volume at every update. **(B)** Average extruded loop length as a function of time for springs with different stiffness $k = 2, 5$ and $8\ \epsilon/\sigma^2$. **(C)** Average extruded loop length as a function of the number of attempted extrusion steps for $\Delta t = 10^2, 10^3$ and $10^4\ \tau_B$, where τ_B is the Brownian time of a DNA bead.

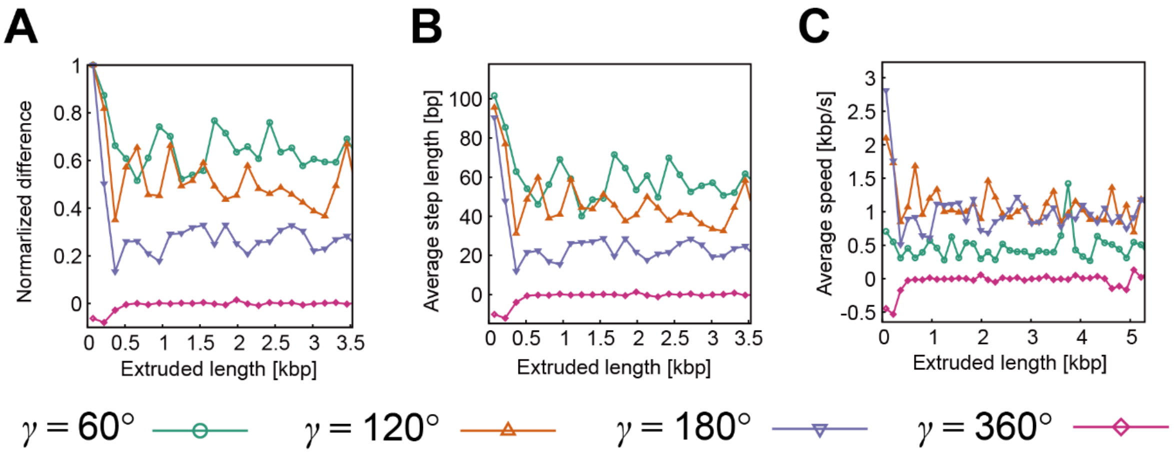

Supplementary Figure 3. Behavior of the model as a function of loop size. (A) Normalized difference, i.e. $(l_{out} - l_{in})/(l_{out} + l_{in})$, of the DNA lengths located outside and inside of the extruded loop within the search range. **(B)** Average step lengths as a function of the extruded length. **(C)** Average extrusion speed as a function of the extruded length. Different colors in the panel correspond to different DNA capture angles.

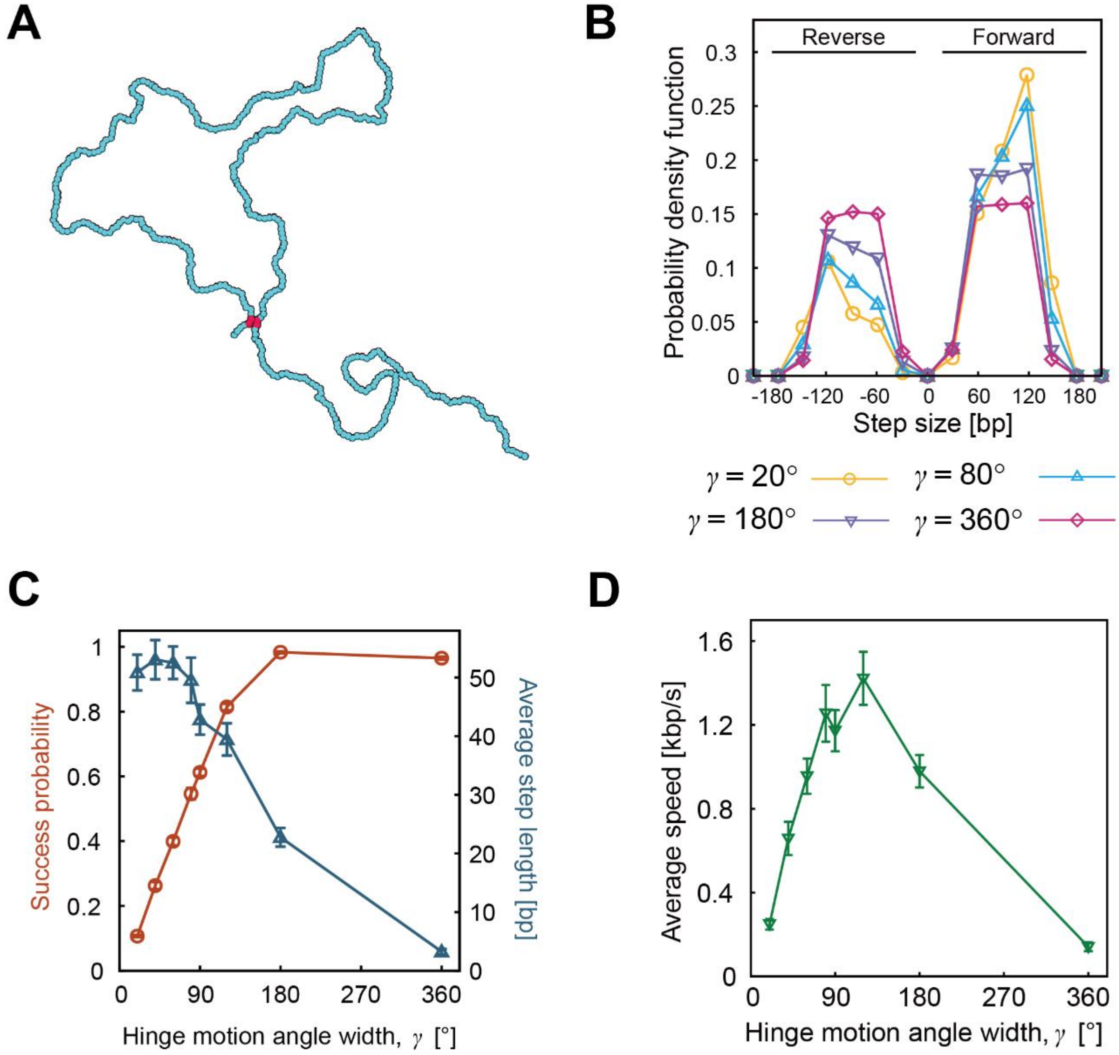

Supplementary Figure 4. Quasi-2D simulations. (A) A snapshot from quasi-2D simulations performed on a polymer confined within a thin slab (thickness $\cong 1\sigma$) . **(B)** Probability distribution of step sizes as a function of the hinge-motion angle, illustrating the forward loop-growth in the quasi-2D case. **(C)** Average step length and success probability as a function of the hinge angle,. **(D)** Average extrusion speed as a function of the search angle, showing an optimal capture angle of 90-120° (compared with 180° seen in 3D simulations).

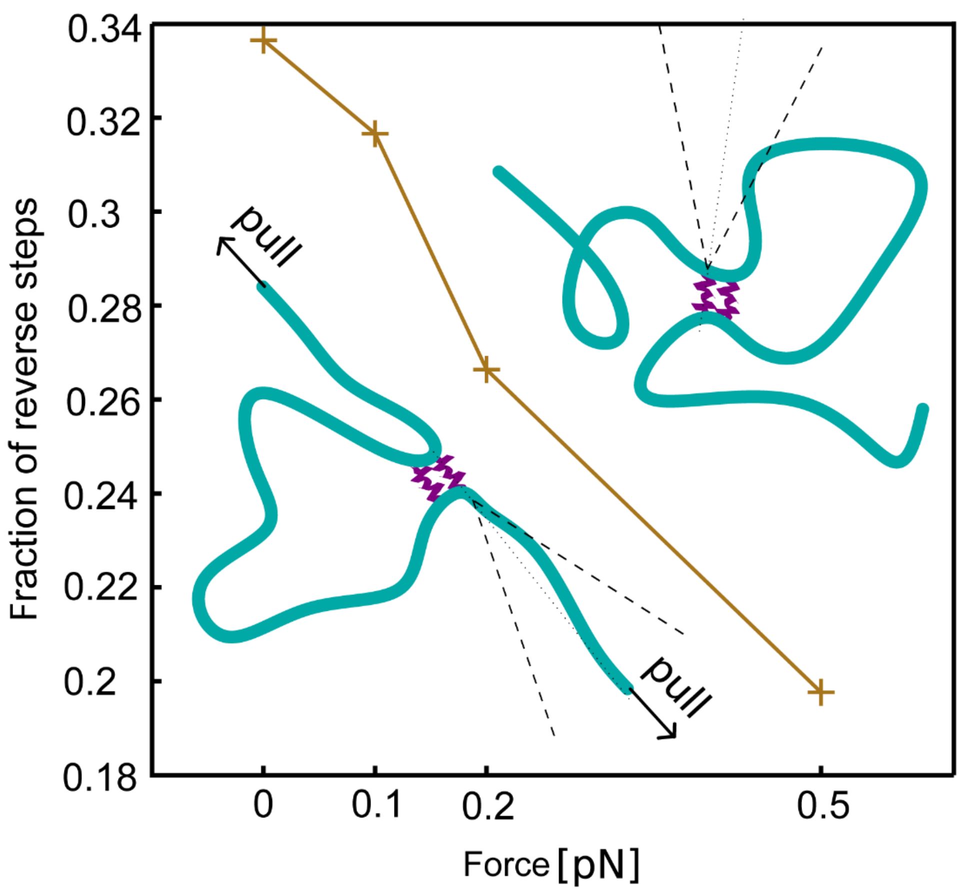

Supplementary Figure 5. Model performance on stretched DNA. Fraction of reverse steps taken by the SMC as a function of pulling force applied to the DNA ends. The inset illustrates two scenarios: an SMC operating on a DNA substrate with free ends (top right) and a DNA substrate under tension (bottom left). The reduced entropy of the tethered assay yields more efficient loop growth and fewer reverse steps.

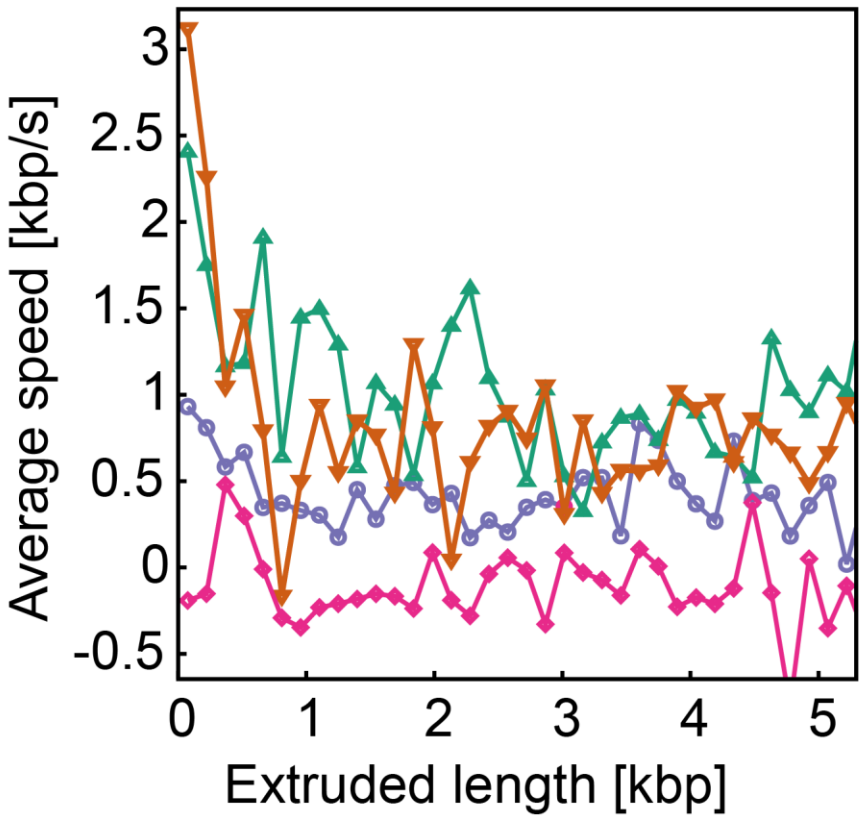

Supplementary Figure 6. Average speed at 1D range limit of 20 beads. In this figure we show the average speed achieved by our SMC model when we extend the 1D range that can be captured by the SMC to 20 beads (instead of 5 beads) and for different angles: green 60, orange 120, blue 180 and pink 360 degrees. The speeds are similar to the ones achieved by the model where the 1D capture range is 5σ.

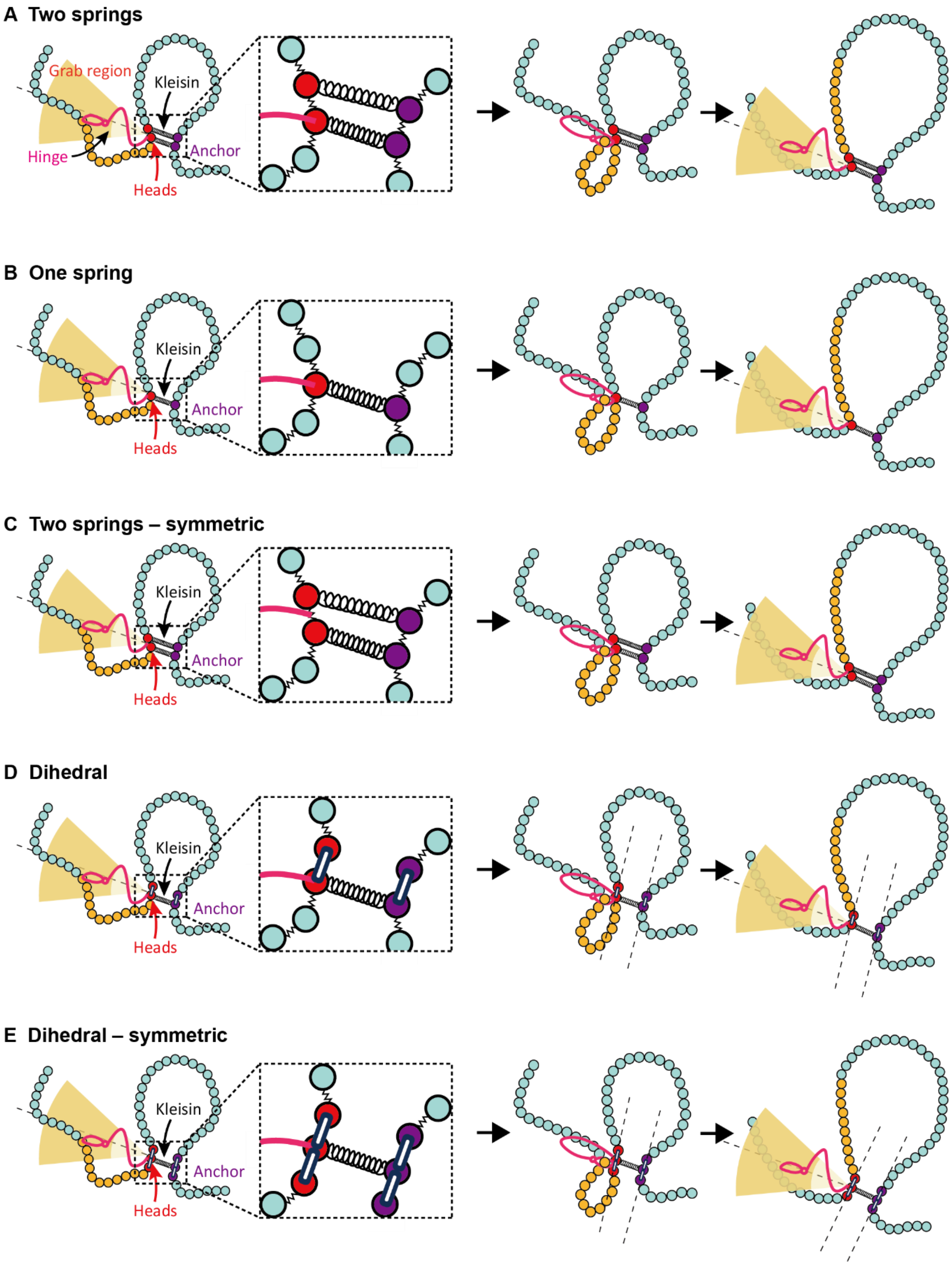

Supplementary Figure 7. Additional model sketches. Step-by-step sketches of different models shown in Figure 4 to simulate local DNA geometric constraints imposed by SMC binding; heads and anchor are denoted with red and purple beads, respectively.

Supplementary video. HS-AFM movie of yeast condensin holocomplex with ATP.

References

1. Shaltiel, I.A., Datta, S., Lecomte, L., Hassler, M., Kschonsak, M., Bravo, S., Stober, C., Ormanns, J., Eustermann, S. and Haering, C.H. (2022) A hold-and-feed mechanism drives directional DNA loop extrusion by condensin. *Science*, **376**, 1087-1094.
2. Lee, B.-G., Merkel, F., Allegretti, M., Hassler, M., Cawood, C., Lecomte, L., O'Reilly, F.J., Sinn, L.R., Gutierrez-Escribano, P. and Kschonsak, M. (2020) Cryo-EM structures of holo condensin reveal a subunit flip-flop mechanism. *Nature structural & molecular biology*, **27**, 743-751.
3. Loeff, L., Kerssemakers, J.W.J., Joo, C. and Dekker, C. (2021) AutoStepfinder: A fast and automated step detection method for single-molecule analysis. *Patterns (N Y)*, **2**, 100256.
